# Hepcidin and Iron Deficiency in Women One Year after Sleeve Gastrectomy: A Prospective Cohort Study

**DOI:** 10.3390/nu13082516

**Published:** 2021-07-23

**Authors:** Thibaud Lefebvre, Muriel Coupaye, Marina Esposito-Farèse, Nathalie Gault, Neila Talbi, Caroline Quintin, Caroline Schmitt, Soumeya Bekri, André Bado, Hervé Puy, Simon Msika, Carole Brasse-Lagnel, Zoubida Karim

**Affiliations:** 1 Centre de Recherche sur l‘Inflammation, Universite de Paris, INSERM, CNRS, 75018 Paris, France; thibaud.lefebvre@aphp.fr (T.L.); muriel.coupaye@aphp.fr (M.C.); neila.talbi@aphp.fr (N.T.); caroline.schmitt@aphp.fr (C.S.); andre.bado@inserm.fr (A.B.); herve.puy@aphp.fr (H.P.); simon.msika@aphp.fr (S.M.); 2 Centre Français des Porphyries, Hôpital Louis Mourier, Colombes, APHP, Nord-Université de Paris, 75018 Paris, France; 3 Service des Explorations Fonctionnelles, Centre Intégré Nord Francilien de l’Obésité (CINFO), Hôpital Louis Mourier, Colombes, APHP.Nord-Université de Paris, 75018 Paris, France; 4 INSERM CIC-EC 1425, Hôpital Bichat, 75018 Paris, France; marina.esposito-farese@aphp.fr (M.E.-F.); nathalie.gault@aphp.fr (N.G.); 5 APHP, Département Epidémiologie, Biostatistiques Recherche Clinique, Hôpital Bichat, 75018 Paris, France; 6 Unité de Recherche Clinique, Hôpital Bichat, AP-HP, Nord-Université de Paris, 75018 Paris, France; caroline.quintin@aphp.fr; 7 Department of Metabolic Biochemistry, Rouen University Hospital, 76000 Rouen, France; soumeya.bekri@univ-rouen.fr; 8 Service de Chirurgie Digestive Oeso-Gastrique et Bariatrique, CHU Bichat, AP-HP, Nord-Université de Paris, 75018 Paris, France; 9 UNIROUEN, INSERM and Rouen University Hospital, Normandy Centre for Genomic and Personalized Medicine, Normandie University, 76000 Rouen, France; carole.lagnel@univ-rouen.fr; 10 Universite de Toulouse INSERM, CNRS, Institut Toulousain des Maladies Infectieuses et Inflammatoires (Infinity), Université Paul Sabatier (UPS), 31000 Toulouse, France

**Keywords:** iron deficiency, obesity, sleeve gastrectomy, hepcidin, iron metabolism, inflammation

## Abstract

Iron deficiency with or without anemia, needing continuous iron supplementation, is very common in obese patients, particularly those requiring bariatric surgery. The aim of this study was to address the impact of weight loss on the rescue of iron balance in patients who underwent sleeve gastrectomy (SG), a procedure that preserves the duodenum, the main site of iron absorption. The cohort included 88 obese women; sampling of blood and duodenal biopsies of 35 patients were performed before and one year after SG. An analysis of the 35 patients consisted in evaluating iron homeostasis including hepcidin, markers of erythroid iron deficiency (soluble transferrin receptor (sTfR) and erythrocyte protoporphyrin (PPIX)), expression of duodenal iron transporters (DMT1 and ferroportin) and inflammatory markers. After surgery, sTfR and PPIX were decreased. Serum hepcidin levels were increased despite the significant reduction in inflammation. DMT1 abundance was negatively correlated with higher level of serum hepcidin. Ferroportin abundance was not modified. This study shed a new light in effective iron recovery pathways after SG involving suppression of inflammation, improvement of iron absorption, iron supply and efficiency of erythropoiesis, and finally beneficial control of iron homeostasis by hepcidin. Thus, recommendations for iron supplementation of patients after SG should take into account these new parameters of iron status assessment.

## 1. Introduction

The prevalence of obesity has increased substantially over the past three decades, reaching 23% in the WHO European Region [1]. Bariatric surgery is recognized as an effective approach for sustainable weight loss in morbidly obese patients. Among bariatric procedures, sleeve gastrectomy (SG) (reduction in the gastric capacity) and Roux-en-Y gastric bypass (exclusion from the gastrointestinal tract) are the most common performed worldwide [2]. However, reports indicate that iron deficiency with or without anemia is frequent in people with obesity and recurrently occurred after each bariatric surgery procedure [3]. In obesity, the adiposity-associated low-grade inflammation, the reduced iron absorption and menorrhagia are the main explanations of iron deficiency [4,5,6]. By reducing the excess of adipose tissue, bariatric surgery should correct the origin of inflammation and restore iron availability. However, postoperative iron deficiency is usually influenced by preoperative iron status, especially in women [7]. The expected causes of this iron deficiency anemia may be the postoperative inflammatory stimulus itself or the reduction in nutrient absorption, and they have a direct impact on iron intake within duodenal enterocytes. Interestingly, the SG, in which intestinal duodenum is preserved, seems to offer less disturbance of the iron balance compared to the Roux-en-Y gastric bypass [8,9]. Nevertheless, the benefit of SG on iron absorption and erythropoiesis remains partially known.

Most of body iron is required for erythropoietic tissues to synthesize hemoglobin in newly formed erythrocytes. The main part of this iron demand (25–30 mg daily) is delivered by reticuloendothelial (RE) iron recycling, a process involving phagocytosis of senescent erythrocytes, proteolysis of hemoglobin and catabolism of heme-by-heme oxygenase 1 (HO−1) in splenic macrophages and Kupffer cells. About 1 mg of iron is lost each day through desquamation of cells from skin and mucosal cells, including the lining of the gastrointestinal tract [10]. In premenopausal adult women, menstruation increases the average daily iron loss to about 2 mg per day, but this loss can be much greater in the case of heavy menstrual bleeding, which is common in obese women [11,12]. Since there is no physiological mechanism of iron excretion, only intestinal absorption adjusts the stores of body iron [13]. Inorganic iron transport by the intestinal microvilli is mostly mediated by the divalent metal transporter DMT1; an integral membrane protein, which imports ferrous iron (FeII) coupled to proton (H^+^) [14,15,16,17]. In macrophages, as well as in enterocytes, iron is exported to the bloodstream through the iron exporter ferroportin (FPN). Hepcidin, secreted by hepatocytes and acting as a hyposideremic factor, is the main regulator of these iron fluxes [18,19]. Hepcidin was first shown in macrophages and HEK cell line, to regulate iron efflux through inhibition of ferroportin [20,21]. However, in mouse intestine, we and others have shown that hepcidin acts additionally on duodenal iron uptake and DMT1 protein expression, possibly leading to efficient inhibition of iron intestinal absorption [22,23].

Hepcidin is regulated by systemic and tissue iron content. In absolute iron deficiency conditions, hepcidin levels are maintained extremely low to increase intestinal iron absorption and iron delivery to erythroid cells. However, hepcidin is stimulated by several inflammatory cytokines, among them the key regulator Interleukin 6 (IL-6) [24]. Thus, inflammatory conditions, such as obesity, can raise the levels of hepcidin, leading to functional iron deficiency which participates to anemia of chronic diseases [25]. 

The laboratory diagnosis of iron deficiency has recently been well established. Several parameters lead to diagnose systemic (serum ferritin, serum iron, serum transferrin, and transferrin saturation levels) and/or erythroid (serum transferrin receptor (sTfR) and erythrocyte protoporphyrin (PPIX)) iron deficiencies. The determination of serum hepcidin levels must complete these analyses and allow us to evaluate the impact of functional iron deficiency that confounds the diagnostic parameters. Hemoglobin level, hematocrit, mean corpuscular volume, and erythrocyte count, are also useful to evaluate iron deficiency anemia. 

In this study, to highlight the eventual benefit of SG on iron absorption and on erythropoiesis, we sought to investigate iron metabolism parameters and the expression of duodenal iron transporters before and one year after sleeve gastrectomy in a cohort of obese women. We used all the diagnostic approaches described above which, to the best of our knowledge, have not been done previously in an obesity context. 

## 2. Patients and Methods

### 2.1. Patients and Setting

The STROBE guidelines were used to ensure the reporting of this observational study [26].

We conducted a multicentric prospective cohort study (NCT01483768) in Bichat (Paris, France) and Louis-Mourier (Colombes, France) University Hospitals. Women aged 18–60 years whose body mass index was greater than 40 kg/m^2^ (or 35 kg/m^2^ when associated with comorbidities), and eligible for SG, were included between September 2012 and October 2014. Women with previous bariatric surgery, past history of hematological disease requiring transfusion, hemochromatosis (transferrin saturation > 45%), Wilson disease, chronic inflammatory disease, elevated transaminases, ongoing treatment by chemo- or radiotherapy for cancer, or those not covered by social security were excluded from the study. Patients, in whom the indication for SG was finally discarded (cancellation of the operation or change of surgical technique), were systematically excluded. Finally, the population of interest corresponded to operated women achieving success of surgery, i.e., a loss of at least 25% of excess weight at one year after surgery [27]. Excess weight was calculated using the measured weight minus ideal weight, determined according to the Metropolitan Life Insurance Company height/weight tables. 

Nutritional guidelines were the same in the two hospital sites. Before surgery, deficiencies including iron deficiency were treated orally if present (with ferrous fumarate or ferrous sulfate). After surgery, a daily multivitamin was systematically prescribed for 1 year and an oral iron substitution was prescribed only if iron deficiency was present before surgery or occurred after surgery. Patients were told to avoid drinking large amounts of tea in case of iron deficiency. Iron substitution was stopped 3 weeks before upper gastrointestinal endoscopy both before and after surgery. 

### 2.2. Endpoints

The main endpoint was to explore the intestinal iron absorption. For that we explored the expression levels of DMT1, FPN and DcytB (Duodenal Cytochrome B reductase) in the duodenum biopsies, and we combined the data with the assessment of iron status. 

The secondary endpoints included serum hepcidin concentration, presence of anemia (defined by serum hemoglobin concentration < 12 g/dL) and its characteristics (microcytic or macrocytic anemia, hemolytic and/or regenerative anemia). In this secondary endpoint, we were interested in exploring the benefit of measuring hepcidin and new markers of erythroid iron deficiency to assess the improvement in iron absorption and erythropoiesis.

### 2.3. Inclusion and Follow-Up

Eligible women were informed at first visit and included after giving written informed consent. Iron supplementation was stopped 3 weeks before the second visit in which gastroscopy with duodenal biopsies and laboratory tests for iron metabolism parameters were performed. The third visit consisted in surgery (SG), and occurred within a year after inclusion. The last visit, 12 to 18 months after surgery, included gastroscopy with duodenal biopsies and laboratory tests for iron metabolism parameters. During each gastroscopy, 6 fragments of duodenal biopsies were collected, and stored frozen for subsequent analysis. Data were collected in a centralized web-based case report form, and related to history of obesity and anthropometric parameters, drug use, usual laboratory tests and iron metabolism parameters.

### 2.4. Hematological and Biochemical Analysis

Serum iron, ferritin, transferrin, CRP and soluble transferrin receptor (sTfR) and alpha-1 acid glycoprotein (AGP) were quantified using the Dimension RXL and Vista 1500 system (Siemens Healthcare, Saint-Denis, France). Transferrin saturation (TS) was calculated as the percent of (serum iron (µmol/L)/serum transferrin (g/L) × 25). The hemoglobin level was measured on an automated counter (Sysmex, Roissy, France), and quantification of IL-6 was performed using a cytometric bead array (BD Bioscience, Le Pont de Cliax, France).

Erythrocyte protoporphyrin was measured as previously described according to the European Porphyria Network guidelines [28].

Serum hepcidin was quantified by the previously published method of LC-MSMS [29].

### 2.5. ARN, RT-qPCR

Total RNAs were extracted from duodenal biopsies using Maxwell^®^ 16 LEV simply RNA Kit (Promega, Charbonnières-les-Bains, France) and performed on the Maxwell^®^ 16 Research System (AS2000) (Promega, Charbonnières-les-Bains, France) according to the manufacturer’s recommendations. The cDNAs were obtained by reverse transcription of 500 ng of total RNAs in a final volume of 20 μL. Real time quantitative PCR was performed in duplicate with the Light Cycler system (Roche Applied Science) using SYBRGreen dye according to the manufacturer’s protocol. Amplification of specific transcripts was confirmed by melting curves profiles generated at the end of the PCR program. The primers were designed with Primer Express software and are DMT1-F:5′-GATGGCAATAGAGCGAGTCAGC-3′; DMT1-R:5′-CAGTTTGTCATGGAGGGATTCC-3′; FPN-F:5′-GGCGTACCCTGTGGTGATG-3′; FPN-R:5′-TGGCATGGGTCTTGCTTTC-3′; Dcytb-R:5′-GTCACCGGCTTCGTCTTCA-3′; Dcytb-F:5′-CAGGTCCACGGCAGTCTGT-3′; RPLP0F:5′-TGCATCAGTACCCCATTCTATCAT-3′; RPLP0R:5′-AAGGTGTAATCCGTCTCCACAGA-3′. Each target gene (DMT1, FPN or Dcytb) was normalized on Ct value for RPLP0 (ΔCt = Ct DMT1 or FPN-Ct RPLP0). The relative amount of DMT1, FPN or Dcytb mRNA levels is given by 2^−ΔΔCt^, where ΔΔCt = [ΔCt of target sample] − [ΔCt of the reference sample].

### 2.6. Extraction Protein, Western Blot

Biopsies from the duodenum were frozen in liquid nitrogen. Samples were disrupted using the TissueLyser LT (QiagenParis, Les Ulis, France) through high-speed shaking (50 Hz) of samples for 2 min in 2 mL microcentrifuge tubes with two 5 mm stainless steel beads and 200 µL of RIPA buffer (20 mM Tris-HCl (pH 7.5), 150 mM NaCl, 1 mM Na_2_EDTA, 1 mM EGTA, 1% NP-40, 1% sodium deoxycholate, 2.5 mM sodium pyrophosphate, 1 mM b-glycerophosphate, 1 mM Na_3_VO_4_, 1 μg/mL leupeptin, 1 mM PMSF and 1% Phosphatase inhibitor cocktail. Samples were then spun down to collect the lysates and protein concentrations were determined by the Bradford assay. Proteins (50 µg) were resolved by TGX Stain-Free 10% gels (Bio-Rad Laboratories, Marnes-la-Coquette, France), and then transferred onto nitrocellulose membrane (Bio-Rad Laboratories, Marnes-la-Coquette, France) for 7 min, 25 V, 2.5 A using the Trans-Blot Turbo system (Bio-Rad Laboratories, Hercules, CA, USA). Membranes were then blocked using PBS 1X containing 5% non-fat milk and 0.05% Tween20, and then incubated with antibodies. The following primary antibodies were used: rabbit FPN antibody (Abnova, Taipei, Taiwan; dilution 1:3000), rabbit DMT1 antibody (Novus biologicals, Lille, France; dilution 1:1000). Membranes were incubated with secondary peroxidase-labelled anti-rabbit (1:5000, Santa Cruz Biotechnology, Dallas, TX, USA) or anti-mouse antibodies (1:10,000, Jackson Immunoresearch Laboratories, WestGrove, PA, USA) and signals were detected with Enhanced chemiluminescence ECL Clarity reagent (Bio-Rad Laboratories, Marnes-la-Coquette, France). To check the equal protein loading, membranes were stripped and re-probed with β-actin antibody (Sigma Aldrich, St. Quentin Fallavier, France). Signals were acquired using a Geldoc™ EZ imager (Bio-Rad Laboratories, Marnes-la-Coquette, France), and quantify with Image Lab (Bio-Rad Laboratories, Marnes-la-Coquette, France). Data were expressed as DMT1 (or FPN)/β-actin protein ratio.

### 2.7. Statistical Analyses

Categorical variables were described by frequencies and percentages; continuous variables by means and standard deviations. Comparisons of continuous variables before and after surgery were performed using paired t tests, or Wilcoxon test, if required. Categorical variables were compared using McNemar’s Chi-squared test for paired samples. Pearson’s correlations were computed between variables.

The significant level of all statistical analyses is a two-sided 5%. 

All statistical analyses will be performed R software (R Foundation for Statistical Computing, Vienna, Austria. http://www.r-project.org/, accessed on 21 June 2021) v. 3.4 or later.

## 3. Results

### 3.1. Characteristics of the SG Patients

**Figure 1:**
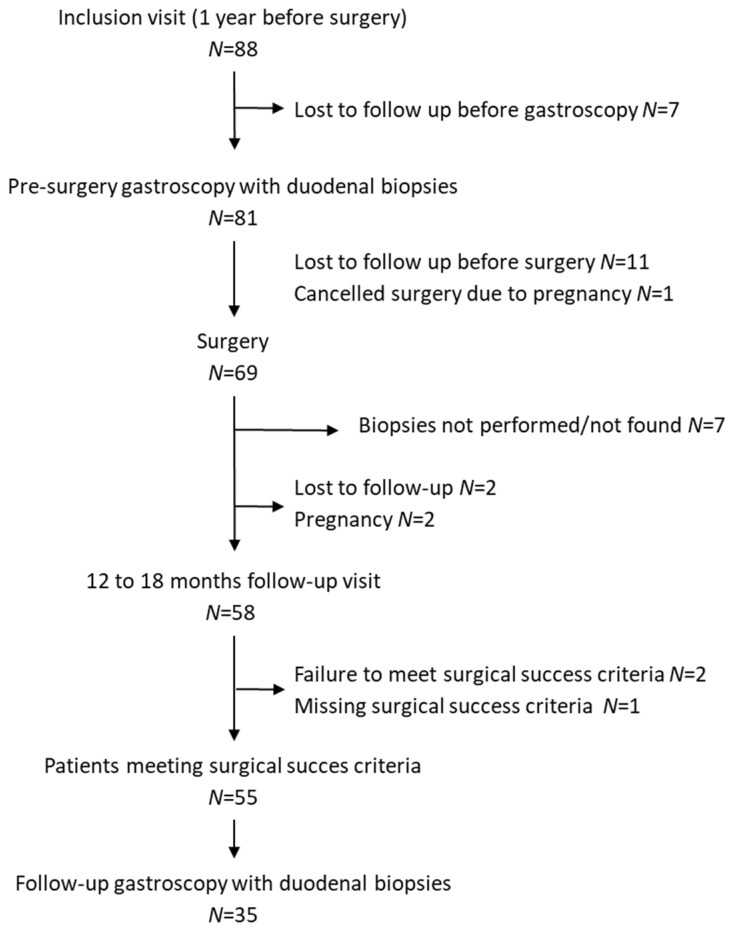
Flow Chart.

The flow diagram of the study is shown in Figure 1, fifty-five women meet the success of surgery criteria. 35 patients had complete follow-up with duodenal biopsies collected before and after SG. Analysis of the anthropometric characteristics at baseline (before surgery), showed high similarity of phenotype with the initial included cohort (*N* = 88) and the other analyzed groups (Table 1). 

The mean age was 37.1 (9.0) years old (Table 1). The mean body mass index (BMI) was 42.1 (5.0) kg/m^2^, but after surgery it dropped to 29.8 (4.7) kg/m^2^, corresponding to a reduction of 12.3 kg/m^2^ (*p* < 0.0001, paired *t*-test). The mean body weight was 112.8 (14.4) kg. Surgery led to a significant weight loss to reach 80.0 (13.5) kg (*p* < 0.0001, paired *t*-test).

### 3.2. Recovery from Systemic and Erythroid Iron Deficiency

The main biological parameters before and after surgery are reported in Table 2 for patients with two available samples. As our study is observational, there was missing data for some biological parameters. However, since their reporting is recommended by the SROBE guidelines, we reported in Table 2 the number of the available data.

**Figure 2:**
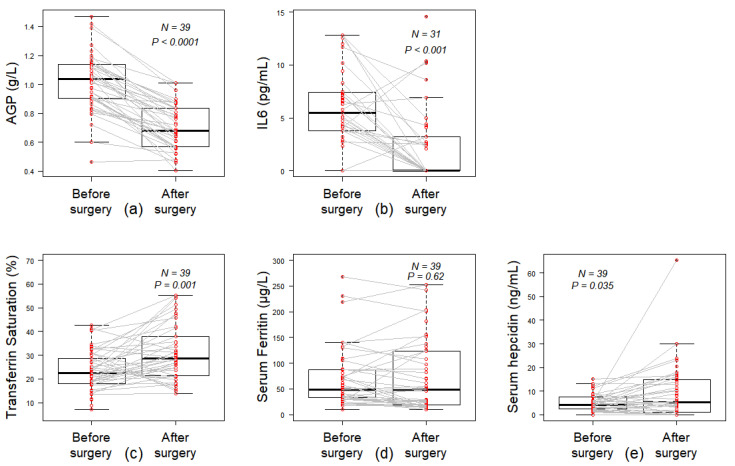
Inflammatory state and iron balance. Paired comparison of pre- and post-operative inflammatory markers values: (**a**) alpha-1 acid glycoprotein (AGP, g/L), (**b**) interleukine-6 (IL6, pg/mL) and iron metabolism markers values: (**c**) serum ferritin (µg/L), (**d**) transferrin saturation rate (TS, %) and (**e**) serum hepcidin concentration. Wilcoxon paired test.

The inflammatory markers AGP and IL-6 levels significantly decreased after surgery (Figure 2a,b). The CRP was positive in 31 out of 49 patients (63.3%) before surgery and it remained positive only in 6 out of 42 patients (14.3%) after surgery (*p* < 0.001). Concentrations of B9 vitamin increased significantly after surgery whereas B12 levels were diminished (Table 2).

The transferrin saturation (TS) rate was at normal range before and after surgery. However, its mean level significantly increased after surgery (Figure 2c). No significant difference was found between the two visits regarding ferritin levels (Figure 2d). However, the concentration of serum hepcidin was increased after the surgery (Figure 2e).

**Figure 3:**
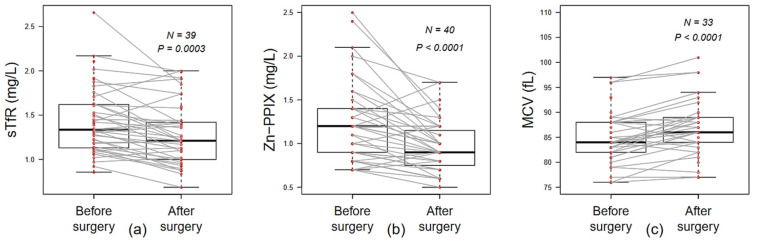
Iron availability for erythropoiesis. Paired comparison of pre- and post-operative erythroid iron-related markers values: (**a**) soluble transferrin receptor (sTfR, mg/L), (**b**) erythrocyte porphyrin (Zn-PPIX, µmol/mmol of red blood cells) (**c**) mean corpuscular volume (MCV, fL). Wilcoxon paired test.

For erythroid iron deficiency markers, both the mean of sTfR and Zn-PPIX levels were found significantly decreased after surgery (Figure 3a,b). This was consistent with significant decrease in the count of reticulocytes and the increase in MCV after surgery (73.5 versus 55.87 G/L, *p* = 0.008 and 85.22 versus 86.59 fL, *p* < 0.001, respectively, Table 2 and Figure 3c). Despite the surgery, hemoglobin levels, red blood cells and hematocrits counts were not significantly different over time (Table 2).

### 3.3. Duodenal Iron Intake Explorations

To evaluate iron absorption from the intestine, we analyzed the mRNA and protein levels of the transporters involved in iron absorption in biopsy specimens from 35 patients undergoing pre- and post-surgery duodenal biopsies. Their biological markers’ evolution are shown in Supplementary Table S1.

**Figure 4:**
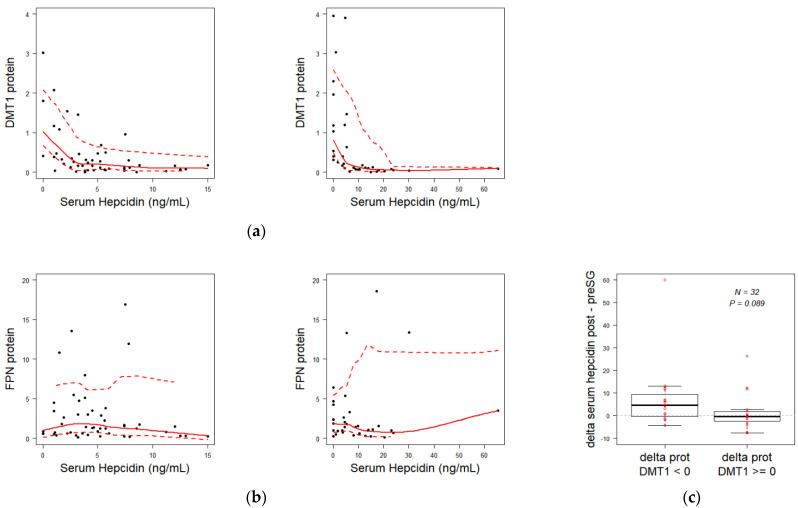
Correlation between serum hepcidin and duodenum protein iron transporters. (**a**) correlation between pre-surgery (left panel) and post-surgery (right panel) serum hepcidin concentration and DMT1 protein (R = −0.045; *p* = 0.001 and R = −0.385; *p* = 0.022, respectively). (**b**) correlation similar to (**a**) for FPN protein (R = −0.117; *p*-value = 0.466 and R = 0.126; *p* = 0.47, respectively). (**c**) comparison of differences in serum hepcidin concentrations after and before surgery between two subgroups of patients: patients whose DMT1 protein decreased after surgery versus patients whose DMT1 protein did not decrease.

Paired comparison of duodenal iron transporters, FPN, DMT1, DcytB did not show significant differences in the mRNA abundance (FPN, DMT1, DcytB) and protein (DMT1 and FPN) (Supplementary data Figure S1). However, circulating hepcidin level was inversely correlated with DMT1 protein level (Figure 4a) but not with FPN abundance (Figure 4b), both before and after surgery. To better elucidate the effect of hepcidin on DMT1 protein, we compared variation rate of hepcidin in SG patients separated into two groups according to a criterion of DMT1 downregulation (17/32 patients) or not (15/32 patients). Patients who developed a downregulation of DMT1 following surgery, had also greater increase in their serum hepcidin level (delta post-/pre-surgery median reaching 4.5 ng/mL), while those who did not show a DMT1 decrease had an unchanged median variation of hepcidin level (close to 0 ng/mL) (Figure 4c).

## 4. Discussion

The most important results of this study are the impact of SG on iron metabolism disorders assessed using biological parameters and the expression of iron transporters in duodenal biopsies. The diagnosis of iron deficiency and anemia has been improved recently. Several inexpensive tools (some already practiced routinely for other explorations) are now available, which make the use of simple markers such as serum ferritin (Ft) or transferrin saturation (TS) insufficient to assess a complete diagnosis of iron status in patients prior iron supplementation. Indeed, Ft and TS value differ depending on context. Both are decreased in absolute iron deficiency anemia, but in functional iron deficiency, TS may be decreased and Ft levels are increased. Here, we used TS, Ft, sTfR, ZnPPIX, hemoglobin, hematocrit, MCV, erythrocyte and reticulocyte counts. Based on these parameters, this study highlights an improvement of iron intake and iron acquisition by erythroid cells in patients undergoing SG with benefic weight loss. Indeed, from a cohort of 55 obese women, we showed that most of them (76.9%) exhibited increased levels of transferrin saturation and MCV with a great decrease in sTFR1 and of Zn-PPIX. We limited the study to women to reduce the heterogeneity of the population and because they represent 80% of obese operated patients [2]. In addition, in our study we chose to focus on premenopausal women who are disposed to iron deficiency due to menstruation and who are, therefore, particularly interesting subjects for exploring the effects of bariatric surgeries [30]. 

In our cohort, we found that patients before surgery did not show severe iron deficiency subsequently to the high preoperative nutritional management of the patients. Indeed, patients were iron supplemented as soon as iron deficiency appeared which must have corrected their iron stores despite morbid obesity. Interestingly, under these conditions, our results showed an endogenous improvement of the martial balance associated with an optimal erythroid iron delivery after SG since for the majority of markers (sTfR, Zn-PPIX and MCV), the normal value shifted from modest to optimal. These results revealed the beneficial effect of SG and weight loss on the iron bioavailability and erythropoiesis. We hypothesized that this improvement is due to the reduction in the inflammation, as testified by the significant decrease in IL6, AGP and CRP. Indeed, chronic inflammation is known to be an important factor for functional iron deficiency and anemia in obese patients and it has been already suggested that bariatric surgery in obese women was linked to reduction in chronic inflammation and to rescue from iron deficiency in these patients [31,32]. In addition, several data show that inflammation directly affects intestinal iron absorption by acting on the expression of the iron transporters DMT1 and FPN [33,34,35]. In addition, inflammation reduces the efficiency of erythropoiesis and stimulates the production of hepcidin which additionally promotes iron retention in tissues stores [24,36]. Unexpectedly, our results showed that hepcidin increased after SG despite the suppression of inflammation. Indeed, hepcidin is an acute phase protein and its production can be induced by multiple stimuli especially IL-6, which is higher in our patients before surgery [37]. Apparently, low iron stores before SG blunt the induction of hepcidin by the low-grade inflammation but after SG, TS was moderately elevated, suggesting re-induction of hepcidin by the improvement of iron availability after SG, independently of inflammation. These results are not surprising because hepcidin is well known to sense serum iron level, and to decrease consequently iron absorption at the level of the intestinal epithelium. Our data additionally figure out the relevance to measure serum hepcidin as a functional marker in the post-operative SG. This measurement must better define the patients requiring oral versus IV iron treatment according to their level of serum hepcidin. Oral iron should be effective for patients with low to normal hepcidin levels, while for patients with high hepcidin levels IV iron would be best recommended.

In our cohort, patients were treated with oral iron unless three weeks before SG to avoid interference with the study. Under these conditions, these patients seem to not suffer from iron deficiency as indicated by their normal iron statue, suggesting that taking care of patients has a beneficial effect on their martial statue.

The negative regulation of iron metabolism by hepcidin is well known. Hepcidin inhibits iron export via interaction with FPN in both enterocytes and macrophages. However, we and colleagues have shown that this interaction can result either in retrieval of FPN from the plasma membrane followed by protein degradation (the case of macrophages and erythroid precursors and probably all unpolarized cells in the body), or simply in inhibition of the activity without internalization (the case of enterocytes [22], and also of red blood cells that are devoid of protein internalization machinery) [38]. In the context of enterocytes, our teams and others have shown that the transport activity and membrane expression of DMT1 were in contrast reduced by treatment with hepcidin of cultured intestinal cells and duodenal loops maintained ex vivo [22]. In this study, we also revealed specific correlation between serum hepcidin and the duodenal DMT1 protein. We were able to define a cut-off of a strong hepcidin action on DMT1 around 10 ng/mL. The recovery of the hepcidin-DMT1 axis may be illustrated by higher rise of hepcidin level when duodenal DMT1 was not increased (Figure 4c), which means that the improvement of iron availability occurs under the control of hepcidin. 

Moreover, we also found no correlation between ferroportin protein abundance and serum hepcidin concentration, suggesting an inhibition of the exporter by mechanisms independent of degradation of the protein, similarly to what was already observed in previous studies [22,38,39]. 

The main limitation of our study was the small size of sample. However, statistical significance was achieved, although a larger sample is needed to clarify the changes over time in hepcidin levels. The comparison of its levels in SG and other surgeries should be investigated. Interestingly, the inflammation was completely abolished after SG and that was associated with great improvement in both iron metabolism and erythropoiesis. These findings generate thoughts about the existence of other biological parameters involved in the regulation of iron metabolism during obesity.

## 5. Conclusions

Our study revealed new light in effective iron recovery pathways after sleeve gastrectomy. This surgery preserves the duodenum, the major site of iron intake. Suppression of inflammation leads to improve iron absorption most probably by increasing DMT1 expression. Iron supply and reduced inflammation promote efficient erythropoiesis. Restored iron stores may finally restore the beneficial control of iron homeostasis by circulating hepcidin, which suppresses duodenal DMT1 when threshold levels are reached. Thus, systematic iron supplementation of patients during the first year after sleeve gastrectomy may not be necessary but long-term biological follow-up are required.

## Figures and Tables

**Table 1:** Anthropometric characteristics of included (*N* = 88), analyzed (*N* = 55) and complete follow-up (*N* = 35) patients with duodenal biopsies.

	*N* = 88	*N* = 55	*N* = 35
Age (year)	37.08 (9.42)	37.09 (9.02)	39.17 (8.23)
Weight (kg)	114.15 (16.42)	112.78 (14.41)	109.74 (11.89)
BMI (kg/m^2^)	42.31 (5.08)	42.08 (5.02)	41.26 (4.36)
Duration of obesity (year)	14.54 (8.79)	14.85 (8.5)	13.96 (7.71)
waist/hip ratio	0.91 (0.11)	0.91 (0.11)	0.89 (0.08)

BMI: Body Mass Index. Data are expressed as mean and standard deviation, m (SD). The duration of obesity was determined according to weight history of the patient, the date of the beginning of obesity corresponding to the first known date with a BMI ≥ 30 kg/m^2^ in adults and BMI Z-score ≥ 2 in children.

**Table 2:** Biological markers’ evolution before and after surgery on the population of 55 patients.

	Before Surgery	After Surgery	*p*-Value	Reference Range
Hemoglobin (g/dL)	12.9 (1.1)	12.6 (0.9)	0.215	11.5–14.9
Red blood cells (T/L)	4.51 (0.35)	4.37 (0.38)	0.118	3.93-5.09
Hematocrit (%)	39.1 (3.0)	38.5 (3.1)	0.447	34.4–43.9
MCV (fL)	85.2 (4.9)	86.6 (6.0)	<0.001	77.9–95.3
Platelets (G/L)	261.7 (44.7)	247.6 (57.1)	0.519	185–445
Leucocytes (G/L)	6.57 (1.76)	5.64 (1.60)	<0.001	4.02–11.42
Folate (μg/L)	6.0 (4.6)	8.7 (5.4)	0.001	3–20
Vitamin B12 (ng/L)	426.6 (162.5)	394.2 (171.7) (171.69)	0.004	189–883
ASAT (U/L)	20.7 (6.4)	18.9 (10.2)	0.152	15–37
ALAT (U/L)	29.9 (10.5)	25.5 (14.1)	0.142	14–59
Gamma GT (U/L)	34.0 (14.1)	34.2 (38.3)	0.326	5–55
ALP (U/100mL)	75.0 (19.0)	69.4 (22.4)	<0.001	50–136
Total Bilirubin (µmol/L)	6.7 (3.3)	9.7 (4.8)	<0.001	0–17
Ferritin (µg/L)	68.7 (55.9)	73.8 (68.6)	0.619	8–252
Transferrin (g/L)	2.55 (0.37)	2.47 (0.42)	0.673	2.00–3.65
Transferrin Iron Binding Capacity	62.7 (9.7)	61.8 (10.5)	0.746	50–80
Transferrin Saturation (%)	23.5 (8.5)	31.2 (11.7)	0.001	20–40
Soluble Transferrin Receptor (mg/L)	1.42 (0.37)	1.27 (0.35)	<0.001	0–1.76
Ceruloplasmin (g/L)	0.29 (0.05)	0.28 (0.07)	0.009	0.2–0.4
Haptoglobin (g/L)	1.18 (0.41)	0.91 (0.34)	<0.001	0.56–2.00

(MCV: Mean Corpuscular Volume; ASAT: Asparate Amino-Trasnferase, ALAT: Alanine Amino-Trasnferase, ALP: ALcaline Phosphatase, TIBC: Transferrin Iron Binding Capacity; sTfR: Soluble Transferrin Receptor. Data is expressed as mean and standard deviation, m (SD).

## Data Availability

The data presented in this study are available on request from the corresponding author.

## Supplementary Materials

The following are available online at https://www.mdpi.com/article/10.3390/nu13082516/s1, Table S1: Biological markers’ evolution before and after surgery on the population of 35 patients with duodenal biopsies. Figure S1: DMT1 and FPN transporters expression in the duodenum before and 1 year after surgery.
