# Supplementary material for: Hepcidin and Iron Deficiency in Women One Year after Sleeve Gastrectomy: A Prospective Cohort Study
Source: Nutrients. 2021 Jul 23;13(8):2516. doi: 10.3390/nu13082516 (PMC8398210; doi:10.3390/nu13082516)
Supplement: Supplementary file 1 [file nutrients-13-02516-s001.zip › nutrients-1288172-supplementary.pdf]

Supplementary data Figure S1

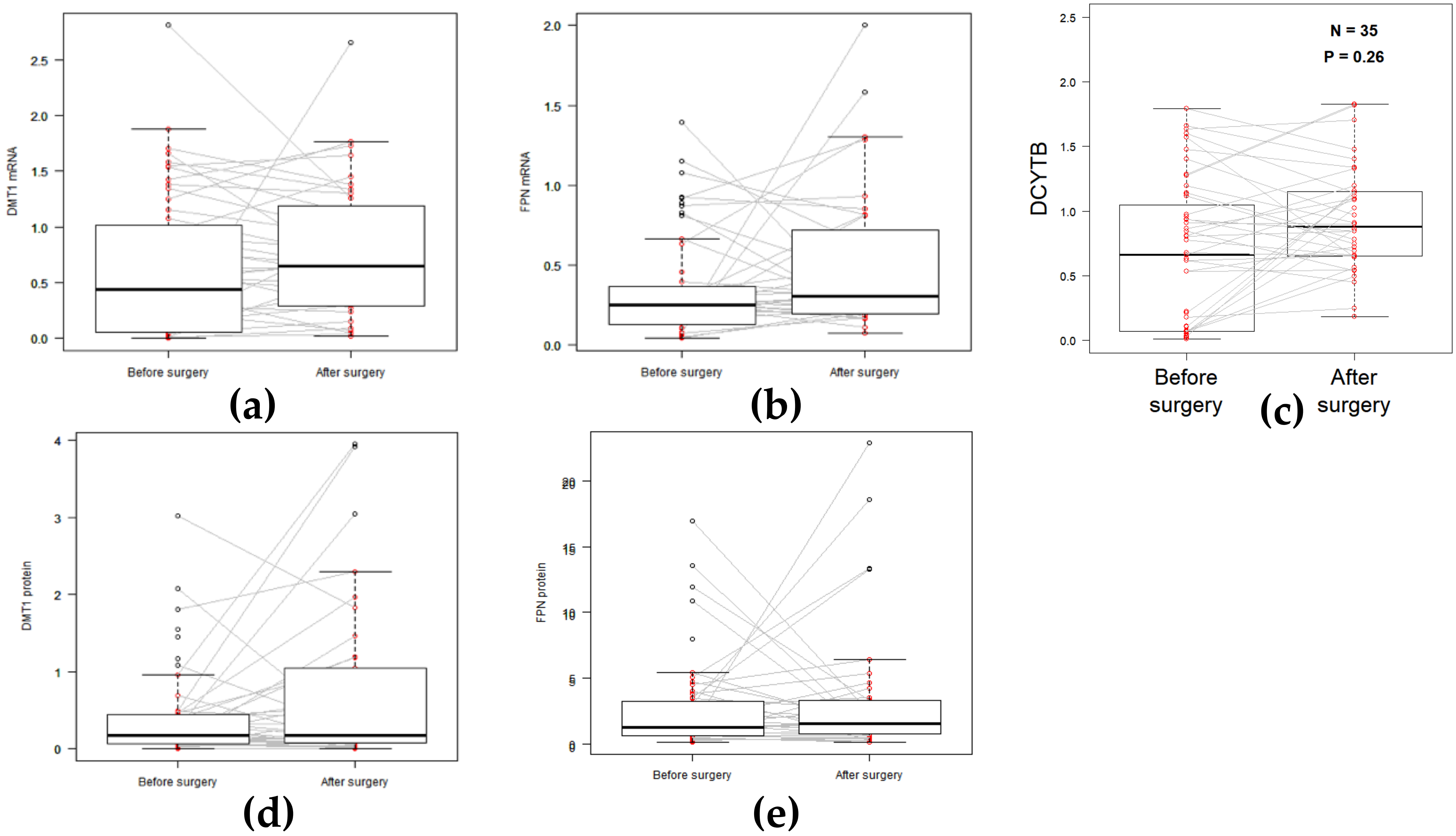

Table S1 ; Population N = 35

	Before surgery	After surgery	p-value	N
Hemoglobin (g/dL)	12.84 (1.17)	12.61 (0.96)	0.61	28
Red blood cells (T/L)	4.45 (0.36)	4.4 (0.4)	0.59	27
Hematocrit (%)	38.45 (3.03)	38.32 (3.2)	1.00	30
MCV (fL)	85.34 (5)	86.7 (6.13)	**5.64×10^{-4}**	28
Platelets (G/L)	256.58 (46.57)	243.07 (56.46)	0.33	26
Leucocytes (G/L)	6.19 (1.53)	5.52 (1.61)	**9.80×10^{-5}**	27
Folate (µg/L)	6.56 (5.3)	8.95 (5.65)	**4.78×10^{-3}**	31
Vitamin B12 (ng/L)	438.12 (167.59)	394.7 (171.17)	0.02	31
ASAT (U/L)	21.12 (6.85)	19.48 (11.26)	0.36	31
ALAT (U/L)	28.67 (11.21)	25.7 (15.79)	0.38	31
Gamma GT (U/L)	35.12 (14.37)	35.24 (38.47)	0.69	31
ALP (U/100mL)	74.82 (21.42)	69.75 (23.91)	**1.66×10^{-4}**	30
Total Bilirubin (µmol/L)	6.93 (3.63)	10.23 (5.09)	**1.28×10^{-4}**	31
Ferritin (µg/L)	72.67 (64.83)	77.12 (70.31)	0.52	31
Transferrin (g/L)	2.47 (0.32)	2.41 (0.39)	0.44	31
Transferrin Iron Binding Capacity	60.62 (8.32)	60.26 (9.7)	0.87	31
Transferrin Saturation (%)	25.74 (7.77)	32.77 (11.63)	**3.88×10^{-3}**	31
Soluble Transferrin Receptor	1.4 (0.37)	1.24 (0.33)	**2.53×10^{-3}**	31
Ceruloplasmin (g/L)	0.29 (0.06)	0.27 (0.07)	**0.04**	28
Haptoglobin (g/L)	1.12 (0.43)	0.89 (0.37)	**8.82×10^{-5}**	30
